# A protocol for characterization of extremely preterm infant gut microbiota in double-blind clinical trials

**DOI:** 10.1016/j.xpro.2021.100652

**Published:** 2021-07-09

**Authors:** Magalí Martí, Johanne E. Spreckels, Maria C. Jenmalm, Thomas Abrahamsson

**Affiliations:** 1 Department of Biomedical and Clinical Sciences, Linköping University, Linköping, Sweden; 2 Department of Genetics, University Medical Centre Groningen, Groningen, the Netherlands; 3 Department of Paediatrics, Linköping University, Linköping, Sweden

**Keywords:** Health Sciences, Clinical Protocol, Sequencing, Microbiology, Molecular Biology

## Abstract

16S rRNA gene sequencing enables microbial community profiling, but recovering fecal DNA from extremely premature infants is challenging. Here, we describe an optimized protocol for fecal DNA isolation, library preparation for 16S rRNA gene sequencing, taxonomy assignation, and statistical analyses. The protocol is complemented with a quantitative PCR for probiotic *L. reuteri* identification. This protocol describes how to characterize preterm infant gut microbiota and relate it to probiotic supplementation and clinical outcomes. It is customizable for other clinical trials.

For complete details on the use and execution of this protocol, please refer to Martí et al. (2021) and Spreckels et al. (2021).

## Highlights

• Customizable protocol for characterization of infant gut microbiota in clinical trials
• Optimized sample preparation for 16S rRNA gene sequencing for infant fecal samples
• Quantitative PCR for probiotic *Lactobacillus reuteri* quantification in infant feces
• Easy-to-use bioinformatic and statistical analysis pipeline

## Graphical abstract

**Figure:**
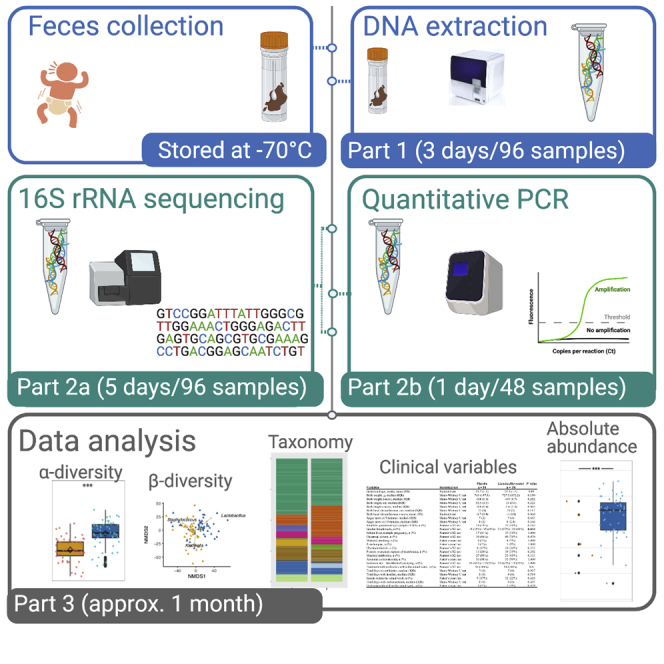

## Before you begin

The 16S rRNA gene sequencing protocol described here is optimized for the study of the gut microbiota of extremely low birth weight (ELBW; birth weight < 1,000 g) extremely preterm (born before gestational week 28+0) infants during the neonatal period, using fecal samples as a proxy. However, we have also used the exact same protocol to study the gut microbiota from full-term infants and from the ELBW infants at the age of two. The optimized protocol is based on the following commercial kits, the QIAmp PowerFecal DNA kit (version 12192013) for DNA isolation and the Illumina protocol “16S Metagenomic Sequencing Library Preparation, Preparing 16S Ribosomal RNA Gene Amplicons for the Illumina MiSeq System” Part # 15044223 Rev. B for 16S rRNA gene sequencing. We recommend reading the original protocols before following the optimized protocol below. In this protocol, 96 samples are multiplexed for sequencing. While we do not recommend multiplexing more than 96 samples, it is possible to multiplex less samples.

The statistical analyses described here are set to investigate the effect of probiotic supplementation on the gut microbiota. As our study is based on two groups (placebo group *vs* probiotic-supplemented group), all the analyses are based on a two-group comparison. However, most of the analyses are applicable to a more than two-group comparison.

### Fecal sample collection and storage

Fecal samples are collected into feces tubes, Screw cap (Sarstedt Cat#80.734.001) as described below. However, we recommend to perform a pilot study to assess the best way for sample collection according to the complexity and possibilities of each study (see Limitations section).

At the neonatal units, feces from newborn infants are collected from the diaper to the feces tube and directly stored at −20°C for short-term storage (up to 24 h) and then placed at −70°C for long-term storage. At home, feces from two years old infants are collected into a feces tube and stored at −20°C (up to one week) until transportation on ice to the laboratory for long-term storage at −70°C.

### DNA mock community

A synthetic mock microbial community (20 Strain Even Mix Whole Cell Material (ATCC® MSA-2002)) is prepared alongside the samples and is used to validate the sequencing quality, comparability between runs as well as, in combination with the negative controls, to determine the prevalence filtering threshold during the data preprocessing step in order to remove potential contaminants. The mock community is provided as a lyophilized pellet and must be dissolved in 1 mL of PBS (not provided), following the manufacturer’s instructions.

### *Lactobacillus reuteri* culture for quantitative PCR standard curves

**Timing: approximately 2 h with an overnight culture incubation**

The quantitative PCR (qPCR) in this protocol is specific for *Lactobacillus reuteri* DSM 17938, which is the probiotic supplemented to the ELBW preterm infants in the studies by Martí et al. (2021) and Spreckels et al. (2021).

A standard curve is prepared from probiotic *L. reuteri* DNA. *L. reuteri* DSM 17938 bacteria were obtained from BioGaia (Stockholm, Sweden) and stored at −70°C. *L. reuteri* DSM 17938 is cultured in Man, Rogosa, and Sharpe (MRS) broth and agar medium, which can be purchased from different suppliers such as Merck Millipore, Oxoid, Sigma-Aldrich, and Thermo Fisher Scientific. DNA from *L. reuteri* cultures is isolated using the EZ1 DNA Tissue kit (Qiagen) on an EZ1 Advanced XL robot (Qiagen).

1. Preparation of MRS agar plates.
  a. Prepare and autoclave MRS medium according to the manufacturer’s instruction.
  b. Pour the medium into bacterial culture plates and cool them at 4°C for 15–20 h (overnight).
  c. Store MRS agar plates at 4°C until use.
2. Preparation of MRS broth medium.
  a. Prepare and autoclave MRS medium according to the manufacturer’s instruction.
  b. Store prepared MRS medium at 4°C.

***Note:*** We recommend preparing the MRS medium one day before starting the *L. reuteri* cultures, so that the medium can cool down over night at 4°C. The MRS medium should have a light brown color.

3. *L. reuteri* cultures on MRS agar plates to obtain a pure culture.
  a. For approximately 5 min partially thaw *L. reuteri* bacteria, until a small portion can easily be taken with a sterile inoculation loop.
  b. With the inoculation loop perform and isolation streak on the MRS agar plates.
  c. Incubate the agar plates at 37°C for 24–72 h.
4. *L. reuteri* cultures on MRS broth.
  a. Pipette 10 mL MRS broth into 2 bacterial broth culture tubes (1 tube for *L. reuteri* and 1 tube as a control).
  b. Pick a single colony from an MRS agar plate and inoculate it into 1 of the tubes with 10 mL MRS broth.
  c. Incubate both tubes anaerobically at 37°C for 24 h.

***Note:*** The control tube is only prepared so that bacterial growth in the tube with inoculated *L. reuteri* can be visually followed and compared to the medium without bacteria. After successful growth of *L. reuteri*, the control tube is used as a counterweight for centrifugation (see DNA isolation from *L. reuteri* cultures below) and can be discarded afterwards. If little bacterial growth is visible in the *L. reuteri* tube, the culture time can be extended.

5. DNA isolation from *L. reuteri* cultures using 2 mL PowerBead Tubes (prefilled with 0.70 mm dry garnet, Qiagen Cat#13123-50) and the EZ1 DNA Tissue kit from Qiagen on an EZ1 Advanced XL robot (Qiagen).
  a. Centrifuge the tube with the *L. reuteri* broth culture at 1,700 × *g* for 10 min at 4°C.
  b. Discard the supernatant without touching the bacterial pellet.
  c. Resuspend the bacterial pellet in 500 μL G2 buffer (from the EZ1 DNA Tissue kit).
  d. Spin down the PowerBead Tubes to ensure that the garnet is at the bottom of the tubes.
  e. Transfer the sample to the PowerBead Tubes.
  f. Lyse bacteria by shaking them on a TissueLyser II for 1 min at 30 Hz.
  g. Transfer 200 μL bacterial lysate to 2 mL sample tubes for automatic DNA extraction on the EZ1 Advanced XL robot.
  h. Start the protocol for purification from bacterial culture samples with an elution volume of 200 μL on the EZ1 Advanced XL robot according to the manufacturer’s instructions.
  i. Store the *L. reuteri* DNA eluate at −20°C.

## Key resources table

**Table:** 

REAGENT or RESOURCE	SOURCE	IDENTIFIER
**Bacterial and virus strains**		

*Lactobacillus reuteri* DSM 17938 for probiotic use	BioGaia AB	https://www.biogaia.com/product-country/product-country-2163/
*Lactobacillus reuteri* DSM 17938 for DNA extraction to generate standards for qPCR runs	BioGaia AB	N/A
DNA mock control ATCC MSA-2002	20 Strain Even Mix Whole Cell Material (ATCC® MSA 2002TM)	https://www.lgcstandards-atcc.org/

**Biological samples**		

Preterm infant stool samples	Martí et al. (2021)	https://doi.org/10.1016/j.xcrm.2021.100206

**Critical commercial assays**		

QIAamp PowerFecal DNA Kit (50 preps)	QIAGEN	Cat#12830-50
16S Metagenomic Sequencing Library Preparation	Illumina	Part # 15044223 Rev. B
Nextera XT Index Kit v 2 (96 indexes, 384 samples)	Illumina	Cat#FC-131-2001
Agencourt AMPure XP, 450 mL	Beckman Coulter	Cat#A63882
2xKAPA HiFi HotStart ReadyMix	Roche	Cat#KK2601
PhiX Control Kit v3	Illumina	Cat#FC-110-3001
MiSeq Reagent Kit v3 (600-cycle)	Illumina	Cat#MS-102-3003
EZ1 DNA Tissue Kit	QIAGEN	Cat#953034
SsoFast^TM^ EvaGreen® supermix	Bio-Rad	Cat#1725201
Qubit dsDNA HS Assay Kits	Thermo Fisher	Cat#Q3285
Ethanol absolute for molecular biology	Sigma-Aldrich	Cat#E7023
Tris-HCl 10 mM, pH 8.5	Roche	Cat#10812846001
PowrBead Tubes, Garnet 0.70 mm	QIAGEN	Cat#13123-50
Screw cap	SARSTEDT	Cat#80.734.001
MRS broth	Merck Millipore	Cat# 69966-500G
MRS agar	Merck Millipore	Cat#69964-500G

**Oligonucleotides**		

16S rRNA primers pair 341F/805R (See 16S Metagenomic Sequencing Library Preparation)	Klindworth et al. (2013)	https://doi.org/10.1093/nar/gks808
LR1/1694 forward primer 5′-TTAAGGA TGCAAACCCGAAC-3′	Romani Vestman et al. (2013)	https://doi.org/10.1159/000347233
LR1/1694 reverse primer 5′- CCTTG TCACCTGGAACCACT -3′	Romani Vestman et al. (2013)	https://doi.org/10.1159/000347233

**Software and algorithms**		

CFX Manager^TM^ Software version 3.1	Bio-Rad	Cat#1845000
bbduk.sh bbmap/38.08	Bushnell (2019)	https://sourceforge.net/projects/bbmap/
FastQC/0.11.5 and MultiQC/1.7	Ewels et al. (2016)	https://github.com/ewels/MultiQC
DADA2 Pipeline Tutorial Dada2 version 1.10.1	Callahan et al. (2016)	https://benjjneb.github.io/dada2/tutorial.html
SILVA database version 132	German Network for Bioinformatics Infrastructure	https://www.arb-silva.de/documentation/release-123/
R Console 3.5.0	The R project for Statistical Computing	https://cran.r-project.org/bin/macosx/
DESeq2 R package version 1.28.2	Love et al. (2014)	https://bioconductor.org/packages/release/bioc/html/DESeq2.html
diverse R package version 0.1.5	Guevara et al. (2016)	https://github.com/mguevara/diverse
phyloseq R package version 1.32.0	McMurdie and Holmes (2013)	https://github.com/joey711/phyloseq

**Deposited data**		

16S rRNA sequencing data set	Martí et al. (2021)	https://www.ebi.ac.uk/ena/browser/view/PRJEB36531
The code for the statistical analysis	Martí et al. (2021)	https://github.com/magge30/PROPEL-ELBW-16S

**Other**		

Placebo (Maltodextrin in oil suspension)	BioGaia AB	N/A
TissueLyser II	QIAGEN	Cat#85300
QIAcube	QIAGEN	Cat#9001292
Qubit Fluorometric Quantification	Invitrogen	Cat#Q33238
Magnetic Stand	Life Technologies	Cat#AM10027
TruSeq Index Plate Fixture Kit	Illumina	Cat#FC-130-1005
EZ1 Advanced XL robot	QIAGEN	Cat No./ID: 9001874
CFX96^TM^ Real-Time PCR Detection System	Bio-Rad	Cat#184-1000, 184-5096
Applied Biosystems™ Applied Biosystems™ 2720 Thermal Cycler	Applied Biosystems	Cat#4359659

## Materials and equipment

Expendable materials including pipette tips, microcentrifuge tubes, and 96-well plates with sealing systems, are not included in the key resources table as this material must be suitable to the local laboratory equipment. In this protocol, the 96-well thermal cycler Applied Biosystems 2720 was used for the Amplicon and Index PCRs in the library preparation protocol for 16S rRNA gene sequencing and CFX96^TM^ Real-Time PCR Detection Systems (Bio-Rad) were used for qPCRs. Additionally, basic equipment of a molecular microbiology laboratory is also needed, including a conventional benchtop microcentrifuge, a benchtop mini centrifuge, a vortex mixer, a heating block, an anaerobic bacteria culture incubator, an autoclave, and glassware for medium preparation.

***Alternatives:*** This protocol uses the QIAcube instrument for a semi-automated purification of DNA, which is compatible with the QIAamp PowerFecal DNA kit. Alternatively, the QIAamp PowerFecal DNA kit protocol can be performed entirely manually.

***Alternatives:*** This protocol uses the QIAxcel instrument to assess the size and integrity of the amplicons. Alternatives are for example the Bioanalyzer (Agilent), the TapeStation (Agilent), or the LabChip (PerkinElmer).

***Alternatives:*** For the qPCR, this protocol uses the SsoFast^TM^ EvaGreen® Supermix and the CFX96^TM^ Real-Time PCR Detection System (Bio-rad). The SsoFast^TM^ EvaGreen® Supermix is also compatible with other thermal cyclers including the Bio-Rad CFX384^TM^ Real-Time System, the Bio-Rad iCycler iQ, MyiQ, and iQ5 Real-Time PCR Systems, or the Roche LightCycler LC480 Real-Time PCR Systems. However, the qPCR settings might need to be adapted for use on different thermal cyclers, see the SsoFast^TM^ EvaGreen® Supermix manual for instructions.

## Step-by-step method details

The protocol and timings below are described for 96 samples, including negative controls, which corresponds to the number of samples multiplexed for the 16S rRNA gene sequencing.

### Part 1: DNA extraction-isolation of total genomic DNA from infant feces

**Timing: approximately 2.5 – 3 h for 12 samples including 1 h automatized (approximately 3 days for 96 samples)**

DNA is isolated using a combination of a first manual step for sample preparation using a modified protocol of the QIAamp PowerFecal DNA kit (version 12192013), followed by an automatized step using the QIAcube Standard Protocol “Purification of DNA from stool and biosolid samples V1” (March 2017). The QIAcube can process 12 samples per run. In the library preparation step, we work with 96 samples at the same time, thus eight extraction batches are required in order to proceed. A negative extraction control (DEPC-treated and filter-sterilized water) should be included for each newly opened QIAamp PowerFecal DNA kit. A mock sample is processed in one of the eight batches for DNA isolation.

1. Thaw fecal samples at 20°C–25°C (room temperature) for approximately 20 min.
2. Add 0.1 grams (± 0.03) of feces to the Dry Bead Tube provided with the kit.

Troubleshooting 1: Insufficient fecal material.

***Note:*** The remaining sample is stored at −80°C for long-term storage.

3. Add 750 μL of Bead Solution to the Dry Bead Tube. Gently vortex to mix.  
4. Add 60 μL of dissolved Solution C1 and invert several times or vortex briefly.
5. Heat the tubes at 65°C for 10 min.

***Optional:*** During the 10 min incubation, prepare the Rotor Adapter according to step 8 in the protocol. This will save you 10 min.

6. Disrupt the samples using a TissueLyser II for 5 min at 30 Hz.
7. Centrifuge the tubes at 13,000 × *g* for 1 min.
8. Prepare the Rotor Adapter as follows:
  a. Position 1: Place a MB Spin Column. Place the lid in position L1.
  b. Position 3: Place the elution tube (1.5 mL). Place the lid in position L3.
9. Inside a fume hood, transfer 450 μL of supernatant into the QIAcube Rotor Adapter position 2.
10. Proceed according to the QIAcube Protocol Sheet. For detailed instructions on how to load the Rotor Adapter, the samples, the reagent bottle rack, the tip racks, and the accessory buffers to the QIAcube, follow the QIAcube User Manual (section 5.3).
11. Start the QIAcube “Purification of DNA from stool and biosolid samples V1” protocol.
12. Once the extraction is finished (approximately 1 h later), the screen will display “Protocol complete”. Follow the instructions given in the QIAcube screen.
13. Proceed directly to the next step for DNA quantification or store the samples at 4°C (short-term storage) or at −20°C (long-term storage).

**CRITICAL:** Cell disruption during DNA isolation influences the microbial community profile. In general, gram-positive bacteria are difficult to lyse; particularly the detection of *Bifidobacteria*, a dominant genus among healthy breast-fed infants, is highly dependent on the mechanical cell disruption during the DNA isolation process (Walker et al., 2015). The original protocol solely uses chemical disruption; we optimized the protocol by adding a mechanical disruption step in order to obtain a more accurate bacterial community profile.

**CRITICAL:** Although the QIAamp PowerFecal DNA kit has recently been indicated as a good kit for gut microbiota profiling (Lim et al., 2020), we recommend to check for kit contamination (kitome), at least once for each new QIAamp PowerFecal DNA kit used. For this purpose, a negative control (i.e., DEPC-treated and filter-sterilized water) is used instead of a fecal sample.

### Part 1: DNA extraction-DNA quantification

**Timing: approximately 1 h for 12 samples**

The DNA concentration is quantified following the manufacturer’s instructions of the Qubit dsDNA HS Assay Kits (MAN0002326 | MP32851, Revision B.0), which uses 1–20 μL of sample for DNA quantification. In Table 1, we provide the exact volumes of the reagents to prepare for the Qubit working solution for the DNA quantification of a 12-samples batch. In Table 2, we provide the exact volumes of reagents, standard, and sample used in this protocol. See Troubleshooting 2 if the DNA concentration is below the detection limit (common for DNA isolated from fecal samples collected during the first two weeks of life of ELBW infants).

**Pause point:** Store the DNA samples at 4°C (short-term storage) until 96 samples have been obtained in order to proceed with the library preparation step.

***Alternatives:*** The preparation of the Qubit working solution as described in Table 1 can be replaced by using the Qubit 1× dsDNA HS (High-Sensitivity) Assay Kit (Thermo Fisher), which provides a ready-to-use reagent and buffer formulation.

**Table 1:** Preparation of the Qubit working solution for DNA quantification of 12 samples

Reagent	Volume (μl/sample)	Volume (μl/12 samples)
Qubit dsDNA HS Reagent	1	12
Qubt dsDNA HS Buffer	199	23884
**Total**	**200**	**2400**

**Table 2:** Preparation of the standards and samples for DNA quantification

Reagent	Standard	Sample
Qubit working solution	190 μL	198 μL
Qubit standard	10 μL	n/a
DNA sample	n/a	2 μL
**Total**	**200 μL**	**200 μL**

### Part 2a: 16S rRNA gene sequencing—library preparation

**Timing: approximately 7–8 h for 96 samples**

The library preparation is based on the Illumina protocol “16S Metagenomic Sequencing Library Preparation, Preparing 16S Ribosomal RNA Gene Amplicons for the Illumina MiSeq System” Part # 15044223 Rev. B. An overview of the protocol is described below with detailed description of the modified steps and otherwise referred to the Illumina protocol.

***Note:*** one 96-well plate contains 93 fecal DNA samples, one mock DNA sample, one work-up negative control from the DNA extraction step, and one no template control from the library preparation.

14. Prepare the Master Mix (Table 3) for the Amplicon PCR.
  a. Transfer 22.5 μL of the prepared Master Mix to each well of a 96-well plate and add 2.5 μL fecal DNA sample, mock DNA, or negative control per well. Pipette up and down several times to ensure proper mixing.
  b. Seal the plate and spin the plate for 30 s to collect the liquid at the bottom of the wells.
  c. Place the 96-well plate in a thermocycler and run the Amplicon PCR program following the settings shown in Table 4.

**Table 4:** Settings for amplicon PCR

PCR cycling conditions			
Steps	Temperature	Time	Cycles
Initial denaturation	95°C	3 min	1
Denaturation	95°C	30 s	30
Annealing	55°C	30 s	
Extension	72°C	30 s	
Final extension	72°C	5 min	1
Hold	4°C	Infinite

**Table 3:** Master mix for amplicon PCR

Reagent	Initial concentration	Volume (μl/sample)	Volume (μl/96 samples)
Amplicon PCR 341F Primer	1 μM	5	500
Amplicon PCR 805R Primer	1 μM	5	500
KAPA HiFi HotStart ReadyMix	2×	12.5	1250
**Total**	**n/a**	**25** (including 2.5 μL of sample)	**n/a**
15. Amplicon PCR clean-up using AMPure XP Beads.
  a. Bring the AMPure XP beads to 20°C–25°C (room temperature) and prepare 80% ethanol by mixing 40 mL of 99.5% ethanol with 10 mL of DEPC-treated and filter-sterilized water.
  b. Follow the Illumina protocol for the PCR clean-up, pages 8–9.
16. Verify that the generated amplicons have the correct size by using a QIAxcel according to the manufacturer’s instructions. The expected amplicon size is ∼550 bp.

Troubleshooting 3: Amplicon PCR results in no amplification product.

**Pause point:** Before proceeding to the Index PCR, the 96-well plate (sealed) can be stored at −20°C for up to one week.

17. Prepare Master Mix (Table 5) for the Index PCR inside a fume hood. Follow the Illumina protocol (pages 10–12) for instructions on how to combine the index primers.
  a. Add 5 μL of clean Amplicon PCR product per well and pipette up and down several times to ensure proper mixing.
  b. Seal the plate and spin the plate for 30 s to collect the liquid at the bottom of the wells.
  c. Place the 96-well plate in a thermocycler and run the Index PCR program following the settings shown in Table 6.

**Table 6:** Settings for index PCR

PCR cycling conditions			
Steps	Temperature	Time	Cycles
Initial denaturation	95°C	3 min	1
Denaturation	95°C	30 s	8
Annealing	55°C	30 s	
Extension	72°C	30 s	
Final extension	72°C	5 min	1
Hold	4°C	Infinite

**Table 5:** Master mix for index PCR

Reagent	Initial concentration	Volume (μl/sample)
Nextera XT Index 1 Primer (N7XX)	1 μM	5
Nextera XT Index 2 Primer (S5XX)	1 μM	5
KAPA HiFi HotStart ReadyMix	2×	25
PCR Grade Water	n/a	10
**Total**	**n/a**	**50** (including 5 μL of sample)
18. Follow the Illumina protocol (pages 13–14) for the Index PCR clean-up.
19. Index PCR size verification.
  a. Dilute the samples 10-fold in a new 96-well plate, by mixing 2 μL of sample with 18 μL of DEPC-treated and filter-sterilized water. This dilution will be used in the following Library normalization step.
  b. Verify that the 10-fold diluted amplicons have the correct size by using a QIAxcel according to the manufacturer’s instructions. The expected amplicon size is ∼630 bp.

Troubleshooting 4: No product after Index PCR.

**Pause point:** Before proceeding to the Library normalization step, the 96-well plate containing the 10-fold diluted samples can be sealed and stored at −20°C for up to one week.

***Note:*** We recommend to also store the non-diluted 96-well plate at −20°C.

### Part 2a: 16S rRNA gene sequencing—library normalization

**Timing: approximately 3.5–4 h for 96 samples**

20. Library normalization.
  a. Quantify the 10-fold diluted libraries (1 library = 1 sample) using the Qubit dsDNA HS Assay (as described above). Quantify each sample at least three times and use the mean.
    ***Note:*** Performing step 20a (DNA quantification) in parallel with step 19 (Index PCR size verification) will save you approximately 1.5 hours.
  b. Calculate DNA concentrations in nM for an average library size of ∼630 bp as follows:
    $$\text{DNA concentration in nM}=\;\frac{\left(\text{concentration in}\frac{\text{ng}}{\text{μl}}\right)x\;10^{6}}{\left(660\frac{\text{g}}{\text{mol}}\right)x\text{ average library size in bp}}$$
  c. Dilute each library to a final concentration of 4 nM, using 10 nM Tris pH 8.5.
  d. Pool 96 libraries into one unique aliquot by combining 5 μL of each normalized library.
  e. Quantify the pooled library with the Qubit dsDNA HS Assay kit (as described above) to ensure its concentration is approximately 4 nM. Do not dilute the pooled library. A concentration of approximately 1.7 ng/μL corresponds to 4 nM.

### Part 2a: 16S rRNA gene sequencing—library denaturing and MiSeq sample loading

**Timing: approximately 2 h sample preparation + 56 h sequencing for 96 samples**

21. Follow the Illumina protocol (pages 17–19) and the suggested volumes in order to obtain:
  a. 20 pM denatured library
  b. 10 pM diluted denatured library
  c. 20 pM denatured PhiX control
  d. 10 pM diluted denatured PhiX control
  e. 20% (v/v) PhiX control spiked-in library. The PhiX control is added in order to increase complexity for the first sequenced bases.
22. Follow the MiSeq System Guide for loading the library.

***Note:*** Use 2 ml screw cap tubes. Five tubes are needed (one per each step mentioned above).

**CRITICAL:** Perform the heat denaturation step of the pooled library with 20% spiked-in immediately before loading the library into the MiSeq reagent cartridge to ensure efficient template loading on the MiSeq flow cell.

**CRITICAL:** Before loading the library, it is very important to invert the Illumina MiSeq cartridge 10 times to mix the thawed reagents. Load the sample (600 μl) into the reservoir Load Samples.

### Part 2b: Quantitative PCR specific for *L. reuteri* DSM 17938

**Timing: 1 day for 2× 41 samples**

Each qPCR run (one 96-well plate) includes five standards, one work-up negative control from the DNA extraction step, one no template control (DEPC-treated and filter-sterilized water), and up to 41 samples in duplicates. The SsoFastTM EvaGreen® Supermix (Bio-Rad) is used for qPCR reactions. The Supermix is a ready-to-use solution containing all reagents except primers and templates necessary for the qPCR. This Supermix can be used with qPCR detection systems like the CFX96^TM^ Real-Time PCR Detection Systems (Bio-Rad), which we used.

For detection of the probiotic *L. reuteri* DSM 17938, we used the primer pair LR1/1694 (Romani Vestman et al., 2013) which generates 177 bp long amplicons of the *L. reuteri* DSM 17938-specific single-copy gene *Lactobacillus reuteri* unknown protein *lr1694* (GenBank accession number: DQ074924.1)

23. Standard curve preparation.
  a. Measure the DNA concentration of the *L. reuteri* DNA eluate using the Qubit dsDNA HS Assay kit as described above. 1 ng *L. reuteri* DNA corresponds to 4.1 × 10^5^ *L. reuteri* bacteria.
  b. Prepare a *L. reuteri* standard stock solution with 5 × 10^5^ *lr1694* gene copies/μL by mixing the *L. reuteri* DNA eluate with nuclease-free water.
  c. Per day, prepare a fresh standard curve with five standards S1-S5 by serially diluting the standard stock solution as shown in Table 7.

**Table 7:** qPCR standard curve

Standard	Preparation		Final concentration (*lr1694* gene copies/μL)	Final concentration (*lr1694* gene copies/reaction)
S1	5 μL standard stock	45 μL nuclease-free water	5 × 10^4^	10^5^
S2	5 μL S1	45 μL nuclease-free water	5 × 10^3^	10^4^
S3	5 μL S2	45 μL nuclease-free water	5 × 10^2^	10^3^
S4	5 μL S3	45 μL nuclease-free water	5 × 10^1^	10^2^
S5	25 μL S4	25 μL nuclease-free water	2.5 × 10^1^	5 × 10^1^
  d. Store the standard stock solution at −20°C and the standards S1-S5 at 4°C until use.

24. Sample dilution.
  a. Dilute fecal DNA samples 10-fold with nuclease-free water.
    ***Note:*** Depending on how many *lr1694* gene copies are present in a sample, the sample can be used undiluted or in a higher dilution. For our study, a few samples had Cq values outside the range of the standard curve. Those samples were re-run in undiluted or 20-fold dilutions and then the Cq values fell within the range of the standard curve.
  b. Store the diluted samples at 4°C until use.

25. Prepare the Master Mix for the qPCR (Table 8).
  a. First, dilute the forward and reverse primers to a concentration of 10 μM using nuclease-free water.
  b. Vortex briefly and spin down.
  c. Add SsoFast^TM^ EvaGreen® Supermix.
  d. Mix by pipetting up and down several times. Do not vortex.

**Table 8:** Master mix for qPCR

Reagent	Initial concentration	Final concentration	Volume (μl/sample)	Volumes (μl/96 samples)
SsoFast EvaGreen® Supermix	2×	1×	10	1000
LR1/1694 forward primer	10 μM	300 nM	0.6	60
LR1/1694 reverse primer	10 μM	300 nM	0.6	60
Nuclease-free water	n/a	n/a	6.8	680
**Total**	**n/a**	**n/a**	**18**	**1800**
26. Preparation of the qPCR plate.
  a. Add 18 μL Master Mix to each well of a 96-well plate.
  b. Briefly vortex and spin down standards S1-S5 and 10-fold diluted fecal DNA samples.
  c. Add 2 μL standard, negative control, or fecal DNA sample per well. Add each standard, negative control, and sample in duplicate.
  d. Seal the plate and spin the plate for 30 s to collect the liquid at the bottom of the wells.
27. Remove the seal and place the 96-well plate in a CFX96^TM^ Real-Time PCR Detection System.
28. Run the qPCR program shown in Table 9.

**Table 9:** Settings for *L. reuteri* DSM 17938-specific qPCR

qPCR cycling conditions			
Steps	Temperature	Time	Cycles
Enzyme activation	98°C	2 min	1
Denaturation	98°C	5 s	40
Annealing and extension	63°C	5 s	
Melting curve	65°C–95°C (0.5°C/step)	5 s/step	1

## Expected outcomes

**Figure 1:**
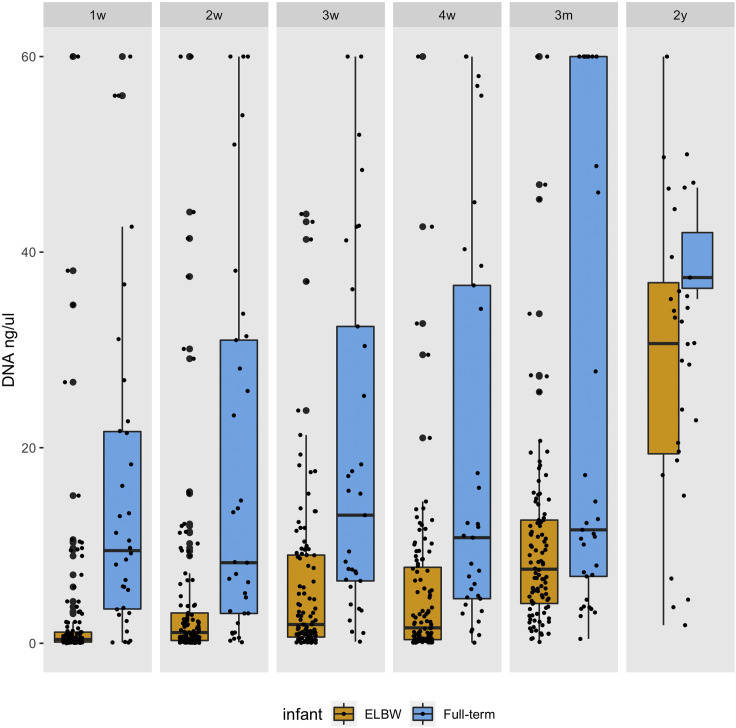
DNA isolation yield (ng/μL) Boxplots (median with 25% and 75% percentiles and 1.5× interquartile range) show the DNA isolation yield from fecal samples collected at different timepoints from extremely low birth weight (ELBW) extremely preterm infants and full-term infants. 1w = 1 week, 2w = 2 weeks, 3w = 3 weeks, 4w = 4 weeks, 3m = post-menstrual week 36 of the ELBW infants and 3 months of the full-term infants, 2y = 2 years.

For a successful sequencing output, the estimated DNA yield after the isolation process must be in the range of 0.05 ng/μL to 60 ng/μL. DNA isolation from fecal samples collected during the first three months of life of ELBW extremely preterm infants commonly results in low DNA yield (Figure 1). The same extraction method applied to fecal samples collected from full-term infants results in higher DNA yield.

The MiSeq system parameters after cycle 25 is completed during the sequencing process are expected to be: Q30 values >70%, Optimal cluster density range 800–1000 K/mm^2^ (acceptable range: 700–1000 K/mm^2^) and Clusters Passing Filter >80%.

After sequencing is completed, demultiplexed .fastq.gz files (a forward and a reverse file per sample) are downloaded from the MiSeq. Sequences are quality-filtered and trimmed, and further processed following the DADA2 Workflow (Callahan et al., 2016) in order to ultimately obtain an amplicon sequence variant (ASV) table containing the reads per each ASV as well as the assigned taxonomy. The ASV table together with the assigned taxonomy and a metadata file form the dataset, which is used for downstream analysis. As an example of expected outcomes, see the original article Martí et al. (2021). The code for the statistical analysis is available at: https://github.com/magge30/PROPEL-ELBW-16S.

After the qPCR is completed, the data is processed using CFX Manager^TM^ Software version 3.1. The expected parameters of the standard curves are listed in Table 10. The Cq values of samples should fall within the range of the standard curve (Table 5), otherwise see Troubleshooting 5. The Cq values between duplicates should not differ more than 0.3, otherwise the qPCR run should be re-run.

**Table 10:** Expected parameters of the qPCR standard curve

Parameter	Mean (95% confidence interval)
Slope	−3.51 (−3.55–−3.47)
y-intercept	42.3 (42.0–42.6)
Efficiency	93% (91%–95%)
R^2^	0.996 (0.995–0.997)
Cq value lower limit of standard curve	24.8 (24.5–25.0)
Cq value higher limit of standard curve	36.4 (36.1–36.7)

Average standard curve values from 24 qPCR runs.

As an example of expected outcomes, see the original articles Martí et al. (2021) and Spreckels et al. (2021).

## Quantification and statistical analysis

### Part 3: Data analysis

As an example of the statistical analysis (part 3), see the original articles Martí et al. (2021) and Spreckels et al. (2021). The code for the statistical analysis is available at: https://github.com/magge30/PROPEL-ELBW-16S. In brief, the analysis pipeline for the amplicon data consists of a first pre-processing step followed by alpha- and beta-diversity analyses in order to characterize the gut microbiota composition in relation to probiotic supplementation and clinical outcomes.

Prior to statistical analyses of the qPCR data, amount of *L. reuteri* bacteria per 1 g wet feces is calculated as follows:

{[ 10ˆ(mean Cq – y-intercept) / slope) ] × dilution factor } / g feces input

The qPCR data is used to determine *L. reuteri* DSM 17938 prevalence and abundance, which can be linked to probiotic supplementation, the gut microbiota composition, and clinical outcomes.

## Limitations

The limitations described below ultimately affect the comparability between studies.

Fecal sample collection is a non-invasive procedure and therefore it is commonly used for the characterization of the human gut microbiota. However, fecal samples are only a proxy for intestinal microbiota. Different parts of the gastrointestinal tract have different physiological conditions resulting in different microbial niches. Thus, when ethically appropriate, the use of gut mucosal biopsies would provide a more representative insight of the intestinal microbiota (Vaga et al., 2020). Tang et al. (2020) recently reviewed the current sampling strategies for human gut microbiota characterization.

Fecal sample collection and storage strategy influence the microbial community profile. In this protocol, fecal samples were recovered from the infant diaper, stored in sterile tubes at −20°C (for up to one week), and subsequently stored at −80°C for long-term storage. Due to the complexity of our study, the days that samples were stored at −20°C varied. Several studies have shown that the microbial composition is affected by the time that fecal samples are stored at different temperatures (e.g., 20°C–25°C (room temperature), 4°C, or −20°C) in combination with or without the addition of DNA stabilizer (Wu et al., 2019, Wang et al., 2018, Panek et al., 2018, Vandeputte et al., 2017, Reitmeier et al., 2020).

The primer choice for generation of 16S rRNA gene amplicons for sequencing will inevitably introduce a bias towards specific taxa (Aloisio et al., 2016, Alcon-Giner et al., 2017, Chen et al., 2019).

A methodological limitation is that 16S rRNA gene sequencing produces compositional data and limits the analysis to relative abundances of bacterial taxa. Another limitation of the 16S rRNA gene sequencing data is that the taxonomical classification is limited in its accuracy to assign lower taxonomic levels like species and strains.

The microbial community profile is further affected by the bioinformatic analyses including, but not limited to, quality-filtering and trimming, which will differ based on the sequencing quality, the method to generate the ASV table, and the reference taxonomical database, which will determine the taxonomical assignations.

A limitation of the qPCR-based detection of probiotic *L. reuteri* in infant feces is that the qPCR detects the presence or absence of DNA, but it does not provide information about whether probiotic bacteria are viable in the infants’ intestines. We recommend cultivating some random samples to verify the viability of the quantified bacteria. In our study, we cultured *L. reuteri* from feces from 16 *L. reuteri*-supplemented infants and verified that the cultures were from the probiotic strain using our strain-specific qPCR method. Fifteen of those 16 samples were positive for *L. reuteri* DSM 17938, even though two of those samples were considered negative in a qPCR performed with DNA extracted from feces without the intermediate culturing step.

The qPCR method described in this protocol is specific for *L. reuteri* DSM 17938, the qPCR protocol must be adjusted for the quantification of other probiotic bacteria used in clinical trials. For adapting our qPCR protocol to detect another probiotic strain, see Troubleshooting 6.

## Troubleshooting

### Problem 1: Insufficient fecal material

It is common that there is insufficient fecal material for feces collected during the first two weeks of life from ELBW extremely preterm infants (step 2).

### Potential solution

To isolate DNA from samples with insufficient material, add 750 μL of Bead Solution (step 3) directly into the tube containing the fecal sample, pipette up and down until all the available material is in the Bead Solution, move it to the tube used for the extraction, and continue the DNA isolation as described.

### Problem 2: Low DNA yield

The concentration of DNA extracted from fecal samples from the first two weeks of life is often too low for quantification (step 13).

### Potential solution

Solution 1: Increase the ratio of DNA / Qubit working solution for quantification by adding 5 μL of DNA into 195 μL of Qubit working solution. Even though up to 20 μL of DNA can be used for quantification with the Qubit assay, we suggest to only increase the sample volume up to up 5 μL because if higher volume is required for quantification, the samples will most likely fail during the library preparation.

Solution 2: If the yield is still below the detection limit after trying Solution 1, perform a second DNA isolation from the same fecal sample and pool the two extractions together, resulting in a final volume of 200 μL. Then concentrate the sample using a vacuum concentrator to evaporate the buffer and resuspend the DNA in 50 μL or 100 μL using the elution buffer from the DNA extraction kit.

### Problem 3: Little amplification product

The Amplicon PCR may result in no amplification product. This occurs often due to too little DNA input, but in some cases, it can occur due to inhibition of the polymerase by substances present in fecal samples (step 16).

### Potential solution

Solution 1: Repeat the Amplicon PCR and at the Amplicon PCR clean-up step using AMPure XP Beads (step 15) elute the purified DNA fragments by adding 27.5 μL (instead of 52.5 μL) of 10 mM Tris pH 8.5 and transfer 25 μL (instead of 50 μL) of the supernatant to a new 96-well PCR plate.

Solution 2: Repeat the Amplicon PCR with 5 μL of DNA sample instead of 2.5 μL and follow the Master Mix instructions in Table 11.

**Table 11:** Alternative master mix for amplicon PCR

Reagent	Initial concentration	Volume (μl/sample)
Amplicon PCR 341F Primer	1.33 μM	3.75 μL
Amplicon PCR 805R Primer	1.33 μM	3.75 μL
KAPA HiFi HotStart ReadyMix	2×	12.5 μL
**Total**	**n/a**	**25 μL** (including 5 μL of sample)

Solution 3: If there is no amplification product after Solution 1 or 2, it could be due to inhibition. Prepare a 10-fold dilution of the fecal DNA sample using DEPC-treated and filter-sterilized water and repeat the Amplicon PCR step using the normal Master Mix conditions (Table 3).

### Problem 4: Lost product after index PCR

The cleaned-up Index PCR results in no product. This is often due to loss of amplification product in the Index PCR clean-up step using AMPure XP Beads (step 19).

### Potential solution

Repeat the Index PCR and run the product on the QIAxcel before preforming the Index PCR clean-up step using AMPure XP Beads (step 18), to confirm there is Index PCR product. This means that the product is lost in the clean-up step (step 18). At the Index PCR clean-up step using AMPure XP Beads (step 18) elute the purified DNA fragments by adding 14 μL (instead of 27.5 μL) of 10 mM Tris pH 8.5 and transfer 12 μL (instead of 25 μL) of the supernatant to a new 96-well PCR plate.

### Problem 5: Cq values fall outside the range of the standard curve

The Cq values of the 10-fold diluted fecal DNA samples should fall inside the range of the standard curve, but sometimes the Cq values fall outside the standard curve range. This can occur due to too low DNA input or due to inhibition of the polymerase by substances present in fecal samples (step 28).

### Potential solution

Repeat the qPCR run using non-diluted or 20-fold diluted fecal DNA instead of 10-fold diluted DNA (step 24). A too high Cq value could be due to too little DNA input or inhibition. Therefore, we recommend testing both options at the same time. A too low Cq value can be increased by using 20-fold diluted fecal DNA.

### Problem 6: To adapt the qPCR protocol for detection of other probiotic bacteria strains

For adapting our qPCR protocol to detect another probiotic bacteria strain other than *L. reuteri* DMS 17938, we suggest considering the following adjustments listed below.

### Potential solution

1. qPCR reaction:
  a. The primer concentration must be adjusted according to the new primer set. The recommended primer concentration range is 300 nM–500 nM.
  b. The sample volume to add into the mix as well as the dilution factor may have to be adjusted. See Troubleshooting 5.
2. qPCR settings:
  a. The annealing temperature must be adjusted according to the primers.
  b. If instead of genomic DNA, plasmid DNA or cDNA is used, the temperature and time for enzyme activation and denaturing must be adjusted. Detailed infomraltion is provided in SsoFast^TM^ EvaGreen® Supermix instruction manual.
  c. The qPCR settings must also be adjusted according to the Real-Time PCR System. Detailed information is provided in SsoFast^TM^ EvaGreen® Supermix instruction manual. In this protocol a CFX96^TM^ Real-Time PCR Detection Systems (Bio-Rad) was used.
3. Standard curve: standards curves can be obtained from cultured bacteria (as indicated in this protocol) and also from plasmids. The detections limits of the standard curve will be specific for each bacteria/plasmid used.

## Resource availability

### Lead contact

Further information and requests for resources and reagents should be directed to and will be fulfilled by the lead contact, Magalí Martí (magali.marti.genero@liu.se).

### Materials availability

This study did not generate new unique reagents.

### Data and code availability

The 16S rRNA sequencing dataset generated during this study is available at the European Nucleotide Archive (ENA) at EMBL-EBI: PRJEB36531 (https://www.ebi.ac.uk/ena/browser/view/PRJEB36531). The code generated during this study is available at: https://github.com/magge30/PROPEL-ELBW-16S.

Infant metadata, qPCR data, and ENA accession numbers are published as supplementary data in Martí et al. (2021).
